# H84T BanLec has broad spectrum antiviral activity against human herpesviruses in cells, skin, and mice

**DOI:** 10.1038/s41598-022-05580-6

**Published:** 2022-01-31

**Authors:** M. G. Lloyd, D. Liu, M. Legendre, D. M. Markovitz, J. F. Moffat

**Affiliations:** 1 Department of Microbiology and Immunology, SUNY Upstate Medical University, Syracuse, NY USA; 2 Division of Infectious Diseases, Department of Internal Medicine, University of Michigan, Ann Arbor, MI USA; 3 Cellular and Molecular Biology Program, University of Michigan, Ann Arbor, MI USA; 4 Cancer Biology Program, University of Michigan, Ann Arbor, MI USA; 5 Program in Immunology, University of Michigan, Ann Arbor, MI USA

**Keywords:** Antiviral agents, Herpes virus

## Abstract

H84T BanLec is a molecularly engineered lectin cloned from bananas with broad-spectrum antiviral activity against several RNA viruses. H84T BanLec dimers bind glycoproteins containing high-mannose N-glycans on the virion envelope, blocking attachment, entry, uncoating, and spread. It was unknown whether H84T BanLec is effective against human herpesviruses varicella-zoster virus (VZV), human cytomegalovirus (HCMV), and herpes simplex virus 1 (HSV-1), which express high-mannose N-linked glycoproteins on their envelopes. We evaluated H84T BanLec against VZV-ORF57-Luc, TB40/E HCMV-fLuc-eGFP, and HSV-1 R8411 in cells, skin organ culture, and mice. The H84T BanLec EC_50_ was 0.025 µM for VZV (SI_50_ = 4000) in human foreskin fibroblasts (HFFs), 0.23 µM for HCMV (SI_50_ = 441) in HFFs, and 0.33 µM for HSV-1 (SI_50_ = 308) in Vero cells. Human skin was obtained from reduction mammoplasties and prepared for culture. Skin was infected and cultured up to 14 days. H84T BanLec prevented VZV, HCMV and HSV-1 spread in skin at 10 µM in the culture medium, and also exhibited dose-dependent antiviral effects. Additionally, H84T BanLec arrested virus spread when treatment was delayed. Histopathology of HCMV-infected skin showed no overt toxicity when H84T BanLec was present in the media. In athymic nude mice with human skin xenografts (NuSkin mice), H84T BanLec reduced VZV spread when administered subcutaneously prior to intraxenograft virus inoculation. This is the first demonstration of H84T BanLec effectiveness against DNA viruses. H84T BanLec may have additional unexplored activity against other, clinically relevant, glycosylated viruses.

## Introduction

Human herpesviruses (HHVs) are enveloped DNA viruses that cause a variety of diseases, ranging from cold sores to skin rashes and infectious mononucleosis [reviewed in^1^]. HHVs cause lifelong infection, and after the initial infection, remain latent until reactivation. HHVs are prevalent worldwide, with up to 95% of the population infected with multiple types^2,3^. Three of the most common HHVs are herpes simplex virus 1 (HSV-1), varicella-zoster virus (VZV), and human cytomegalovirus (HCMV). HSV-1 is an alphaherpesvirus that causes oral, ocular and genital infections in approximately half the U.S. population, however its prevalence has declined slightly in the last 15 years^4^. VZV is another alphaherpesvirus that causes varicella (chicken pox) upon initial infection and herpes zoster (shingles) upon reactivation. Fortunately, introduction of a vaccine has reduced the yearly incidence of varicella in the US by over 80%^5^, although globally the incidence remains much higher. Finally, HCMV, a betaherpesvirus, is the leading cause of viral congenital birth defects and can cause life-threatening complications in immunocompromised patients, such as those with transplants^6,7^. HCMV is endemic, and prevalence ranges from 40 to 70% in industrialized nations to 100% in developing countries^8,9^.

The need for improved antivirals is urgent, with an ever pressing need to develop broad-spectrum treatments. The nucleoside analogues acyclovir, cidofovir, and ganciclovir inhibit the DNA polymerase and are active against all HHVs in culture, yet they are only approved for clinical use against one or a few viruses^10^. Unfortunately, serious side effects, such as neurotoxicity, renal failure, and bone marrow suppression, have been associated with many of these antivirals^11–13^. Although these antivirals are effective, their use is limited, and resistance can develop with continued treatment in immunocompromised people. This highlights the need for new treatments that are less toxic and that have a broad range of activity across many virus families.

One emerging strategy is the use of lectins to bind glycoproteins on the viral envelope [reviewed in^14^]. Naturally occurring lectins have been explored as broad-spectrum antimicrobials to inhibit viruses, bacteria, and other microbes. One promising lectin, BanLec, was derived from a banana lectin^15^, and proved an effective strategy to combat HIV^16^. Unfortunately, wild-type BanLec was a T-cell mitogen [reviewed in^17^] and activated basophils and mast cells^18^. To address these issues, we developed H84T BanLec, replacing histidine at position 84 with threonine, which markedly reduced mitogenicity while maintaining the antiviral properties of the lectin^15^. H84T BanLec is effective against hepatitis C virus (HCV)^15^, human immunodeficiency virus (HIV)^15,19,20^, influenza A and B viruses (IAV, IBV)^15,21^, and Ebola virus^22^. More recently, computer modeling showed that H84T BanLec is expected to bind the SARS-CoV-2 S-glycoprotein with high affinity^23^. Wild-type BanLec is active against additional pathogens, including bovine viral diarrheal virus (BVDV) and bovine alphaherpesvirus 1 (BoHV-1)^24^ and *Salmonella*^25^. Interestingly, when a change similar to that which converted BanLec to H84T BanLec was made in the related Malaysian banana lectin, producing F84T Malay BanLec, broad-spectrum antiviral activity was retained and mitogenicity was reduced in a manner comparable to H84T BanLec^26^.

The mechanism of action for H84T BanLec is virus dependent. Broadly, H84T BanLec binds to high-mannose N-glycans on the viral envelope to block attachment and entry, particularly in the case of HIV^15,19^. H84T BanLec had minor effects on IAV attachment, mainly blocking fusion of the viral envelope in the endosome^21^. H84T BanLec affects Ebola virus entry as well as downstream steps in the virus life cycle^22^. So far, there have been no observed effects on assembly, budding or release. H84T BanLec has the properties of a broad-spectrum antiviral, potentially treating both RNA and DNA viruses through multiple mechanisms that have in common binding to the surface glycoproteins of the individual viruses.

High-mannose N-linked glycans are present on the envelope proteins of many viruses, including human herpesviruses. N-linked glycosylation sites are found on many HSV-1 glycoproteins^27–34^, VZV glycoproteins^35–37^, and HCMV glycoproteins^38–41^. Moreover, it is likely that more glycosylation sites on herpesvirus envelope glycoproteins have yet to be identified. While wild-type and H84T BanLec are effective against many different viruses, H84T BanLec, which due to its greatly decreased mitogenicity has greater therapeutic potential than does wild-type BanLec, had not been tested against human herpesviruses. Based on prior studies and the presence of N-linked glycans on the viral envelopes, we expected H84T BanLec to have antiviral activity against several HHVs. To address this hypothesis, we evaluated the effectiveness of H84T BanLec in preventing viral spread using cell and tissue cultures infected with VZV, HCMV, or HSV-1, and found that H84T BanLec was effective in a dose dependent manner, as well as when treatment was delayed. We also evaluated the effects of H84T BanLec on VZV in vivo, using an athymic nude mouse model engrafted with adult human skin (NuSkin mouse model) and found that it was effective as a prophylactic treatment. H84T BanLec prevented viral spread in cells and in infected human skin explants.

## Results

### H84T BanLec evaluation in cultured cells

**Figure 1:**
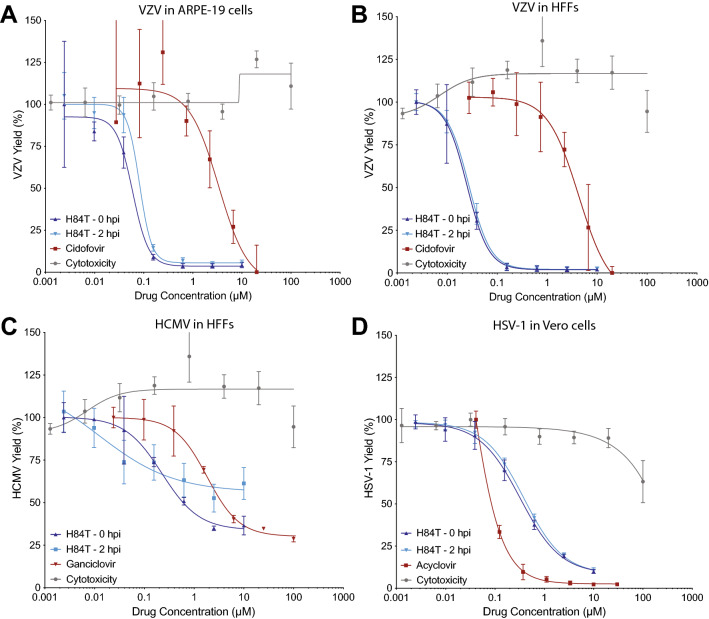
H84T BanLec potency against VZV, HCMV, and HSV-1 in cultured cells. Cell-free viruses were added to cell monolayers at 37 °C, and H84T BanLec or positive control antiviral compounds were added at 0 h post-inoculation (HPI) or 2 HPI. The antiviral compounds remained in the culture medium until virus yield was measured by bioluminescence imaging. ARPE-19 cells or HFF cells were infected with VZV for 3 days (**A**, **B**), HFF cells were infected with HCMV for 7 days (**C**), and Vero cells were infected with HSV-1 for 2 days (**D**). Virus yield was calculated from the average Total Flux of untreated wells divided by the average Total Flux of the treated wells. H84T BanLec cytotoxicity was measured using a neutral red dye uptake assay in Vero cells treated for 48 h (**D**) and HFFs or ARPE-19 cells treated for 72 h (**A**–**C**). Each point represents the mean ± SD. N = 3–6 biological replicates.

H84T BanLec was evaluated against VZV, HCMV, and HSV-1 in cell culture assays. Antiviral activity and cytotoxicity were evaluated in HFFs (human foreskin fibroblasts) and ARPE-19 (adherent retinal pigmented epithelial) cells for VZV, in HFFs for HCMV, and Vero cells for HSV-1. Confluent HFFs and ARPE-19 cells or sub-confluent Vero cells were infected with virus, and H84T BanLec was added immediately after infection. Virus and H84T BanLec remained on the cells for the duration of the experiment. Antiviral activity was assessed by bioluminescence imaging at 2 days post infection (DPI) for HSV-1, 3 DPI for VZV, and 7 DPI for HCMV, based on their different replication rates in cells. The use of bioluminescence to detect changes in viral load was previously validated^42^. The antiviral activity and subsequent 50% effective concentration (EC_50_) of all compounds was calculated as changes in bioluminescent signal, or Total Flux, as previously described^43^. H84T BanLec was highly effective against all three viruses with no cytotoxic effects in the cells up to 100 µM (Table 1, Fig. 1). For VZV, HCMV, and HSV-1, the EC_50_ values were in the sub-micromolar range with very high selective indices.

**Table 1:** Efficacy of H84T BanLec against human herpesviruses in cultured cells.

Virus	Cell type	EC_50_ (µM)	CC_50_ (µM)	SI_50_*
VZV	HFF	0.025	> 100	4000
	ARPE-19	0.059	> 100	1695
HCMV	HFF	0.23	> 100	441
HSV-1	Vero	0.33	> 100	308

*SI (selectivity index) calculated as CC_50_/EC_50_.

The mechanism of action of H84T BanLec is virus-dependent, and it is thought to act primarily on attachment, entry, and/or, in the case of Ebola virus, also on replication^15,19,21,22^. Thus, the timing of treatment may be important for the effectiveness of H84T BanLec. We investigated the timing of H84T BanLec treatment against human herpesviruses in cultured cells by starting treatment at the time of virus infection or 2 h post-infection (HPI) to allow for virus attachment. Once added to the cells, virus and H84T BanLec were present in the media for the duration of the experiment. Bioluminescence was measured on the same timetable described above. For VZV and HSV-1, there was no difference in the EC_50_ of H84T BanLec if treatment was administered at the time of infection or delayed (Fig. 1A, B VZV; Fig. 1D HSV-1). For HCMV, there was a noticeable right shift to the EC_50_ when treatment was delayed (Fig. 1C). When treatment was initiated at the time of HCMV infection, the EC_50_ was 0.23 µM, whereas the EC_50_ could not be determined when treatment was delayed 2 h. This suggests that H84T BanLec antiviral activity was lost when treatment was delayed.

### Effects of H84T BanLec on virus life cycle in cultured cells

**Figure 2:**
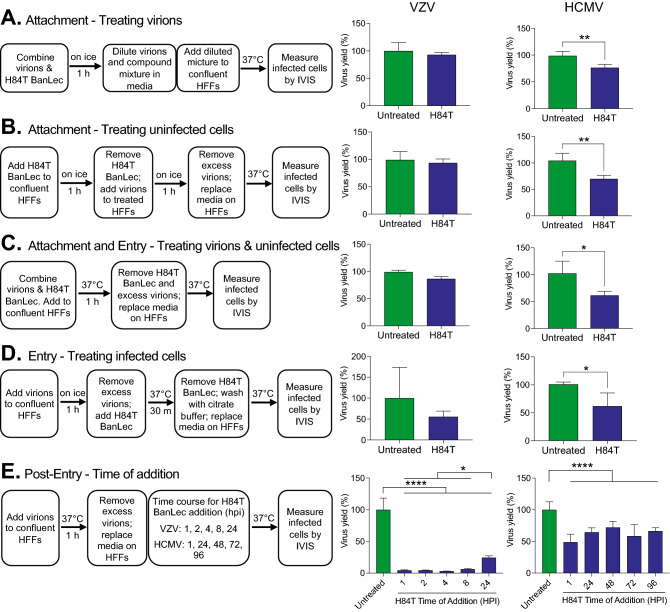
Effects of H84T BanLec on extracellular VZV and HCMV virions and on infected cells. Each experimental condition tests a different aspect of H84T BanLec’s mechanism of action including attachment (**A**, **B**), attachment and entry (**C**), entry (**D**), and post-entry steps (**E**). Cell-free virions and H84T BanLec were added to HFF cell monolayers based on the experimental design for each condition. Cell-free VZV was prepared from a fresh culture of infected HFF cells; the MOI was approximately 0.01. HCMV was prepared from a frozen, titered stock; the MOI was 0.05. H84T BanLec was used at a concentration of 100 nM for VZV and 2 µM for HCMV. (**A**) Virions were mixed with H84T BanLec on ice for 1 h, and were then diluted 1:50 for VZV and 1:1000 for HCMV prior to adding to HFFs. (**A**–**D**) Infected cells were measured by bioluminescence imaging 24 h post-infection for VZV, and 7 days post-infection for HCMV. (**E**) Infected cells were measured by bioluminescence imaging 48 h post-infection for VZV, and 7 days post-infection for HCMV. Virus yield (%) was calculated from the average Total Flux of untreated wells divided by the Total Flux of each well. Bars and error bars represent the mean + SD. N = 4 biological replicates. Asterisks indicate significance [*p < 0.05, **p < 0.01, ****p < 0.0001, *p* < 0.05, Student’s t-test (**A**–**D**) or one-way ANOVA with Dunnett’s post hoc test (**E**)].

The evaluation in cultured cells suggested that H84T BanLec works differently against alphaherpesviruses (HSV-1 and VZV) and betaherpesviruses (HCMV), with the latter likely affected at the stage of attachment and/or entry, and the former at post-entry steps. To address this possibility further, H84T BanLec was combined with virions or infected cells at different phases of the virus life cycle. VZV and HCMV were selected for these experiments because they grow in the same cell type, HFFs, and so the results could be directly compared. The process for each experiment is displayed in Fig. 2. Cell-free virus and confluent HFFs were used for all experiments, and the concentration of H84T BanLec was 100 nM for VZV and 2 µM for HCMV. H84T BanLec had no effect on the infectivity of cell-free VZV, and it did not prevent attachment or entry (Fig. 2A–D). In contrast, H84T BanLec significantly reduced the infectivity of HCMV virions by approximately 50% (Fig. 2A, Student’s t test, *p* = 0.0046). Similarly, H84T BanLec reduced HCMV attachment and entry by half when it was added to the culture medium during the extracellular phase (Fig. 2B–D, Student’s t test, *p* < 0.05).

To address post-entry events, time-of-addition studies were performed (Fig. 2E). The timing of treatment and virus yield measurements was based on the length of the virus life cycle, which is approximately 16 h for VZV and 96 h for HCMV. H84T BanLec added to VZV-infected cells at 1, 2, 4, or 8 h post inoculation significantly lowered virus yield at 48 h to approximately 5% of the untreated cultures (*p* < 0.0001, one-way ANOVA, Dunnett’s post hoc test). Additionally, H84T BanLec reduced VZV yield when it was present from 24 to 48 h to about 20% of the untreated cultures (*p* < 0.05, one-way ANOVA, Dunnett’s post hoc test). The difference in VZV yield between early timepoints and the 24 h timepoint reflects the typical cell–cell spread of VZV from one infected cell to approximately 5 adjacent cells on day 1 and to about 20–25 cells on day 2. H84T BanLec added to HCMV-infected cells (without an overlay or neutralizing antibody to prevent dispersal of nascent virions) at 1, 24, 48, 72, or 96 h post inoculation significantly lowered virus yield by half after 7 days in culture (*p* < 0.0001, one-way ANOVA, Dunnett’s post hoc test). Taken together, H84T BanLec had disparate effects against VZV and HCMV. These results suggest that H84T BanLec reduced the infectivity of HCMV virions by half and prevented VZV cell–cell spread.

### H84T BanLec evaluation in skin organ culture

**Figure 3:**
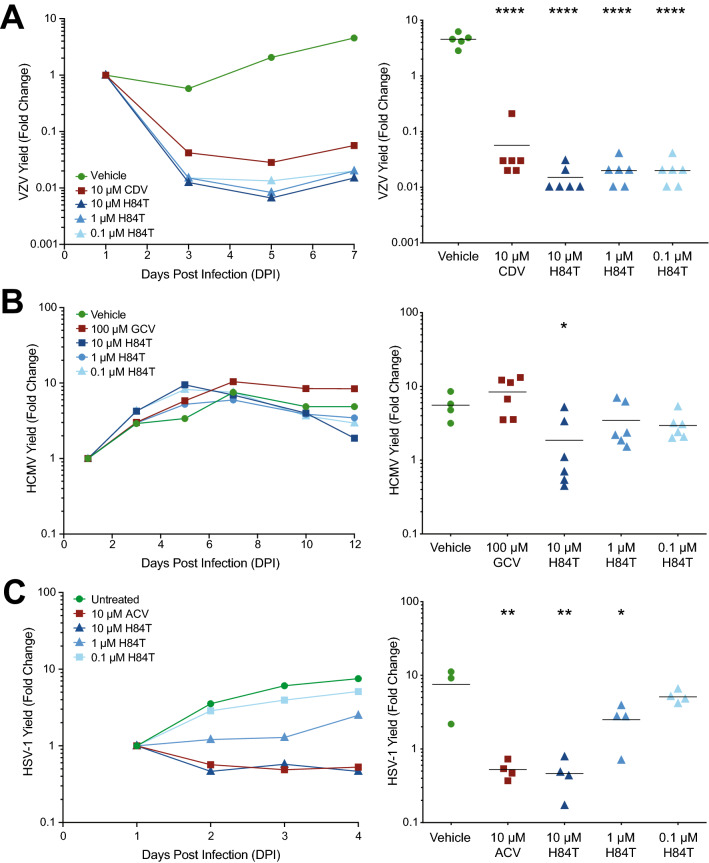
H84T BanLec evaluation in skin organ culture. Adult skin explants were inoculated with VZV (**A**), HCMV (**B**), or HSV-1 (**C**) and then placed on NetWells over medium that contained H84T BanLec or positive control antiviral compounds (cidofovir, CDV, 10 µM; ganciclovir, GCV, 100 µM; acyclovir, ACV, 10 µM). Virus spread was measured by bioluminescence imaging starting on 1 DPI. Virus yield on subsequent days was calculated by dividing the average Total Flux (photons/sec/cm^2^/steradian) by the average Total Flux on 1 DPI. Viral growth kinetics (left panels, each symbol is the average for the group) were analyzed for statistical significance on the last day (right panels), where each symbol represents one piece of skin and the bar is the mean of the group. Asterisks indicate significance between the treated groups and vehicle, [*p < 0.05, **p < 0.01, ****p < 0.0001, *p* < 0.05, one-way ANOVA, Dunnett’s (A, C) or Dunn’s (B) post hoc test]. N = 4–6 biological replicates.

H84T BanLec was highly potent against VZV, HCMV, and HSV-1 in cell culture, so we further investigated its effects using an adult skin organ culture (SOC) model. Recently, we established this model for VZV and HCMV^44^, and it was adapted to study HSV-1 here. Whole thickness adult skin was thinned with a skin grafting knife and cut into approximately 1-cm^2^ pieces^44^. Skin pieces were inoculated with VZV or HSV-1 by scarification, or with HCMV by intradermal injection, then placed at the air–liquid interface on NetWells, and H84T BanLec was added to the underlying culture medium at the time of inoculation. H84T BanLec concentrations were informed by the EC_50_ values in cell culture, the complexity of adult human skin, and our experience performing antiviral studies in skin tissue. Virus growth and spread was detected by bioluminescence imaging and yield was calculated as fold change of Total Flux (photons/sec/cm^2^/steradian). The groups included: untreated, positive control compounds [cidofovir (CDV), 10 µM; ganciclovir (GCV), 100 µM; acyclovir (ACV), 10 µM], and H84T BanLec at three concentrations (10, 1, and 0.1 µM). As expected, the viruses grew with normal kinetics in the untreated groups and the positive control compounds prevented virus spread (Fig. 3). For VZV-infected skin, all concentrations of H84T BanLec prevented virus spread (Fig. 3A, *p* < 0.05, one-way ANOVA, Dunnett’s post hoc test). In HCMV-infected skin, only the highest H84T BanLec concentration, 10 µM, reduced virus spread (Fig. 3B, *p* < 0.05, one-way ANOVA, Dunn’s post hoc test). As expected, GCV was not effective in this assay because GCV protects infected cells from the lytic effects of HCMV replication, allowing the bioluminescent signal to persist. In HSV-1-infected skin, a dose-dependent response was observed, where 10 µM H84T BanLec reduced virus spread the most while 0.1 µM H84T BanLec had no effect (Fig. 3C, *p* < 0.05, one-way ANOVA, Dunnett’s post hoc test). The 1 µM concentration also reduced HSV-1 spread, but to a lesser extent. Overall, 10 µM H84T BanLec limited spread of all three viruses in SOC.

### Delayed H84T BanLec addition prevents virus spread in skin organ culture

**Figure 4:**
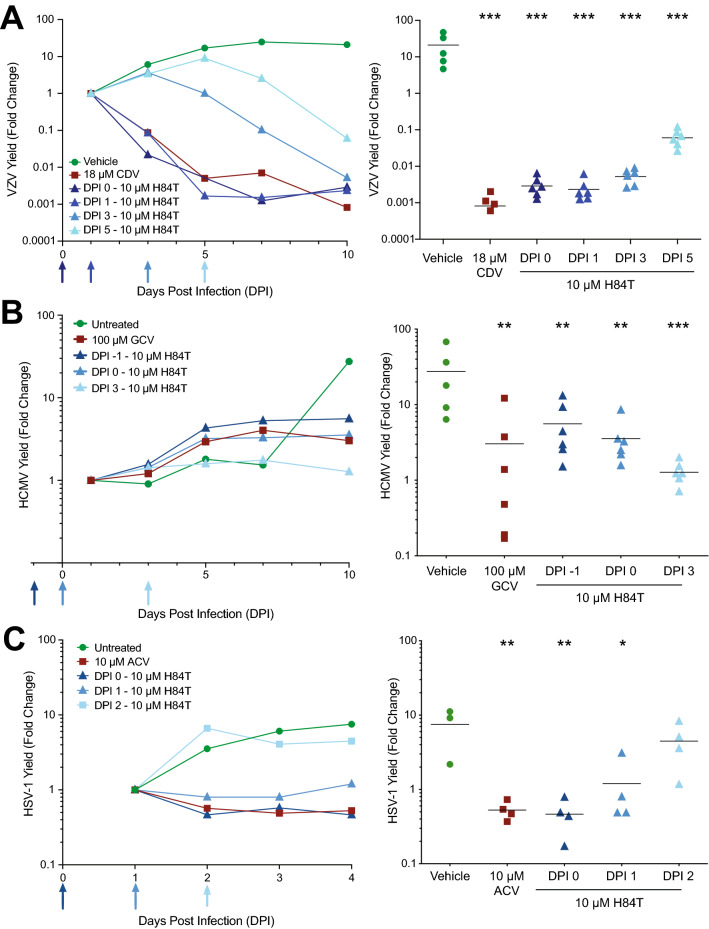
H84T BanLec addition after infection prevented VZV, HCMV, and HSV-1 spread in skin. Adult skin explants were inoculated with VZV (**A**), HCMV (**B**), or HSV-1 (**C**) and then placed on NetWells over medium that contained H84T BanLec (10 µM) added according to the schedule on the *x*-axis (blue arrows). Control antiviral compounds (cidofovir, CDV, 18 µM; ganciclovir, GCV, 100 µM; acyclovir, ACV, 10 µM) were added at DPI 0. Virus spread was measured by bioluminescence imaging starting on DPI 1. Virus yield on subsequent days was calculated by dividing the average Total Flux (photons/sec/cm^2^/steradian) by the average Total Flux on DPI 1. Viral growth kinetics (left panels, each symbol is the average for the group) were analyzed for statistical significance on the last day (right panels), where each symbol represents one piece of skin and the bar is the mean of the group. The Vehicle and ACV groups in (**C**) are the same as in Fig. 3C, as these experiments were conducted together. Asterisks indicate significance between the treated groups and vehicle (*p < 0.05, **p < 0.01, ***p < 0.001). *p* < 0.01, one-way ANOVA, Dunnett’s post hoc test. N = 4–6 biological replicates.

Antiviral treatment for HSV-1 and VZV infections begins after lesions appear in skin, whereas HCMV is often treated prophylactically in immunocompromised patients. It was not known if H84T BanLec would prevent virus spread in skin after infection was established. Results in cultured cells suggested that timing of treatment may alter the effectiveness of H84T BanLec, however such studies are also necessary in skin. Viruses have slightly different replication cycles and can behave differently due to the skin’s microenvironment. Skin was prepared and infected as described above, and 10 µM H84T BanLec was added to the culture medium at different times pre- or post-inoculation, depending on the replication rates of each virus. In all assays, the positive control compounds were added at the time of inoculation (DPI 0) and significantly reduced virus spread (Fig. 4, *p* < 0.01, one-way ANOVA, Dunnett’s post hoc test). When H84T BanLec was added to VZV-infected skin at DPI 0, 1, 3 or 5, there was a significant downturn in VZV yield as soon as it was added (Fig. 4A, *p* < 0.01, one-way ANOVA, Dunnett’s post hoc test). When H84T BanLec was added to HCMV-infected skin at DPI -1, 0, or 3, virus spread was significantly reduced (Fig. 4B, *p* < 0.01, one-way ANOVA, Dunnett’s post hoc test). When H84T BanLec was added to HSV-infected skin at DPI 0, 1, or 2, it only lost effectiveness when added at 2 DPI (Fig. 4C, *p* < 0.01, one-way ANOVA, Dunnett’s post hoc test). Here, we showed that either delaying treatment or pre-treating with H84T BanLec is an effective strategy, which suggests that it could be effective as a prophylactic or therapeutic treatment against HHVs.

### Effects of H84T BanLec on production of infectious virions in skin

**Figure 5:**
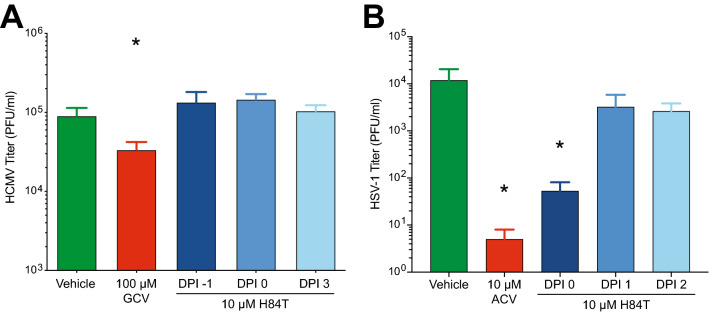
H84T BanLec reduces the infectivity of HSV-1 but not HCMV in skin organ culture. HCMV and HSV-1 infected skin pieces from the assay shown in Fig. 4 were collected on the last day and processed for virus titration. (**A**) HCMV was measured by TCID_50_ assay in HFF cells (*p < 0.05, one-way ANOVA with Dunnett’s post hoc test). (**B**) HSV-1 was measured by plaque assay in Vero cells with a methyl cellulose overlay. (*p < 0.05, one-way ANOVA with Dunnett’s post hoc test). Bars and error bars represent the mean + SEM. N = 3–6 infected skin pieces per group.

Virion assembly and egress are late events in the herpesvirus replication cycle, and understanding how antivirals affect virus replication is important in evaluating their overall effects. To investigate the effects of H84T BanLec on virion production, we used skin infected with HCMV-fLuc-eGFP or HSV-1 R8411 from the time-of-addition assays presented above and collected it on DPI 14 or DPI 4, respectively. (VZV-infected skin was not collected because infectious VZV virions are not isolated from skin in a quantitative manner.) HCMV or HSV-1 virions were extracted from skin pieces and then infectivity was measured by TCID_50_ assay or plaque assay, respectively. As expected, ganciclovir significantly reduced HCMV titers (Fig. 5A, *p* < 0.05, one-way ANOVA with Dunnett’s post hoc test) and acyclovir significantly reduced HSV-1 titers (Fig. 5B, *p* < 0.05, one-way ANOVA with Dunnett’s post hoc test). H84T BanLec had no effect on HCMV titers, regardless of when treatment was initiated (Fig. 5A), supporting the results presented in Fig. 2. H84T BanLec also had no effect on HSV-1 titers when treatment was delayed by 1 or 2 days (Fig. 5B), which is longer than the replication time of HSV-1. H84T BanLec reduced HSV-1 titers when H84T BanLec was added at the time of infection (Fig. 5B, *p* < 0.05, one-way ANOVA with Dunnett’s post hoc test). Interestingly, these results suggest that H84T BanLec may act on HSV-1 assembly and egress. This assay does not distinguish between input virus inoculum, which may persist in the dense skin tissue, and nascent virions. These results support H84T BanLec having different effects on the life cycle of each virus and should be further explored.

### H84T BanLec is not toxic to skin

**Figure 6:**
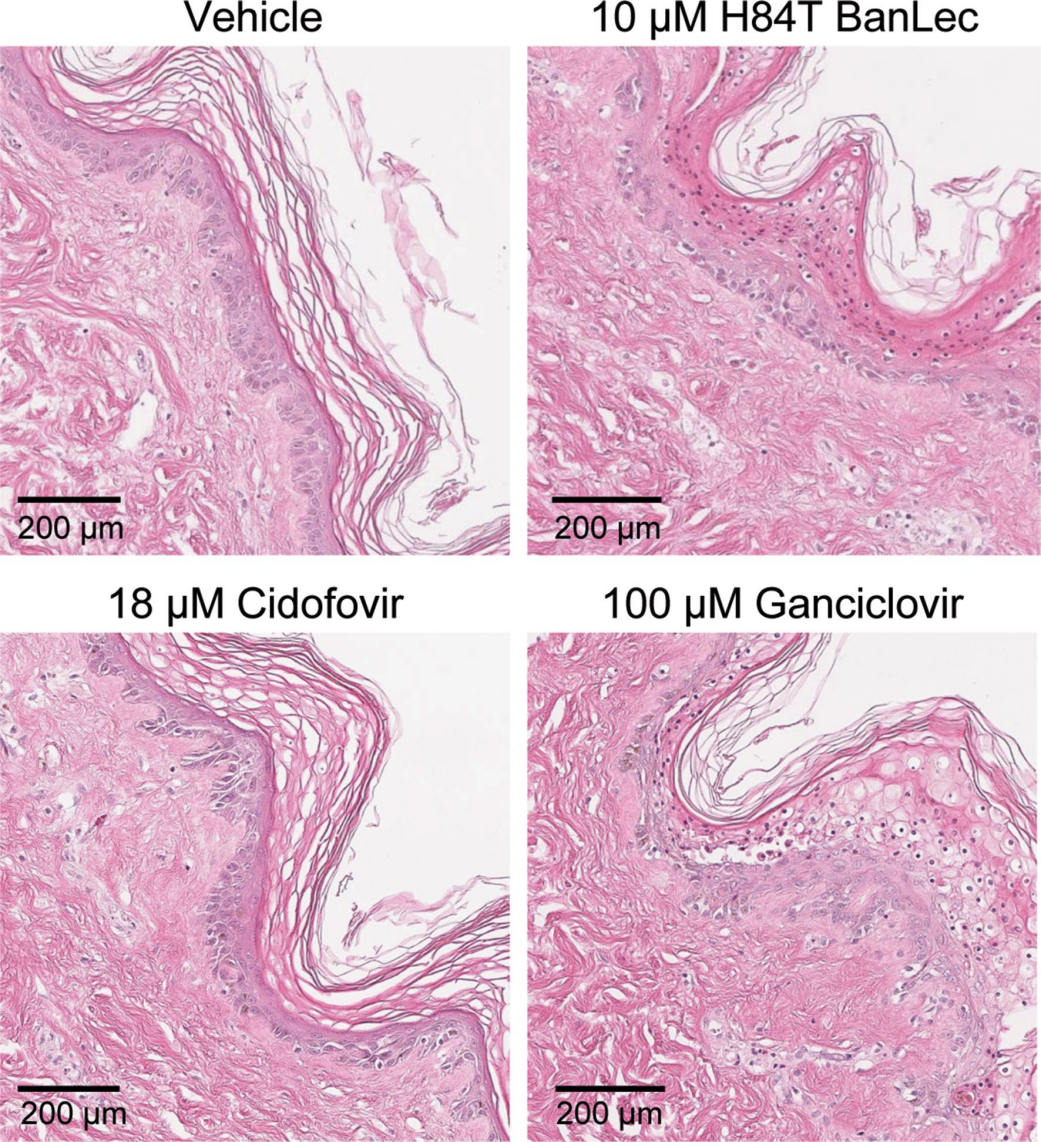
Effects of H84T BanLec on adult skin. Skin was infected with HCMV, cultured on Netwells, and treated every other day with compounds in the medium. Skin was collected 14 DPI and fixed in 4% paraformaldehyde for H&E staining. The epidermis faces the right side of the image (appears purple) and the dermis is to the left (appears pink). The epidermis appears normal, and the dermal–epidermal junction is intact (HCMV replicates in dermal cells). Images are representative of 2–3 sections per tissue sample from a single tissue donor.

Although H84T BanLec was not cytotoxic, its effects on whole human skin were unknown. Skin infected with HCMV was selected because the 14-day treatment regimen lasted the longest. Skin was infected with HCMV-fLuc-eGFP and H84T BanLec was added to the culture medium. The groups included: untreated, 18 µM cidofovir, 100 µM ganciclovir, and 10 µM H84T BanLec. After 14 days, skin pieces were collected and fixed in formalin for paraffin-embedding and H&E staining. Histological analysis showed healthy skin tissue, with an intact dermis, epidermis, and dermal–epidermal junction in all groups (Fig. 6). This indicates that H84T BanLec was non-toxic to human skin tissue, even with extended exposure.

### Efficacy of H84T BanLec in the NuSkin mouse model

**Figure 7:**
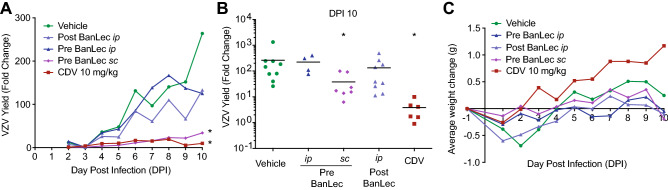
H84T BanLec evaluation in NuSkin mice infected with VZV. NuSkin mice were implanted with a single subcutaneous xenograft of adult human skin and xenografts were inoculated 3–4 weeks later with VZV by intraxenograft injection and scarification. Mice were treated with 50 mg/kg/day H84T BanLec by subcutaneous (*sc*) or intraperitoneal (*ip*) routes given 6 h pre-inoculation (−6 HPI) and on DPI 4 and 8 to the Pre-BanLec groups, or by the *ip* route on DPI 3 and 7 to the Post-BanLec group. Cidofovir was administered daily by *ip* injection from DPI 3–9. (**A**) VZV yield was measured by bioluminescence and the fold change calculated as the Total Flux on each day divided by the Total Flux on DPI 2 or 3 (the lowest value for that mouse). Lines represent the average VZV yield per group. (**B**) VZV yield on DPI 10 was analyzed for statistical significance where each symbol represents one mouse, and the bar is the mean of the group. (*) Signifies statistical significance between the vehicle and a treatment group (*p* < 0.05, one-way ANOVA, Dunnett’s post hoc test). (**C**) Mouse weights were measured daily, and the lines show the average weight change for each group from the onset to conclusion of the study. N = 4–12 mice per group.

While it was not known whether H84T BanLec was effective against HHVs in vivo, we expected that it would be well-tolerated in mice based on previous studies^15,21,22^. One virus was selected, VZV, because the selective index was the highest (approximately 4000) and the mouse model for evaluating antiviral compounds is well established^43^. A mouse model for testing antiviral activity against HCMV exists^45,46^, but it is complex and uses human fetal tissue. Multiple mouse models exist for HSV-1 antiviral testing, but these are not approved in our laboratory. Therefore, we evaluated the effects of H84T BanLec on VZV replication in the NuSkin mouse model, which we developed previously and employ often^44^. Athymic nude mice were implanted with adult human skin tissue subcutaneously above the left flank. After 3–4 weeks, xenografts were infected by scarification and direct injection with VZV-ORF57-Luc. Virus spread was monitored by daily bioluminescence imaging. The groups included: vehicle, cidofovir (10 mg/kg/day), and three different treatment regimens with H84T BanLec (50 mg/kg/dose). The group labeled Pre BanLec *sc* received H84T BanLec subcutaneously as a prophylactic treatment 6 h before infection (-6 HPI) and again on DPI 4 and 8. Subcutaneous injections were administered between the shoulder blades. The group labeled Pre BanLec *ip* received H84T BanLec intraperitoneally as a prophylactic treatment 6 h before infection (-6 HPI) and again on DPI 4 and 8. The group labeled Post BanLec *ip* received H84T BanLec intraperitoneally as a therapeutic treatment on DPI 3 and 7. Cidofovir, given daily from DPI 3–9 intraperitoneally, significantly reduced virus spread, as expected (Fig. 7A, B, *p* < 0.05, one-way ANOVA, Dunnett’s post hoc test). Interestingly, only the prophylactic, subcutaneous H84T BanLec treatment regimen effectively reduced virus spread (Fig. 7A, B, *p* < 0.05, one-way ANOVA, Dunnett’s post hoc test). While there was a trend toward reduced virus spread in the intraperitoneally treated groups (Pre and Post BanLec), the results did not reach significance. The compounds were well-tolerated with no significant weight loss or adverse drug effects observed in either the cidofovir or H84T BanLec groups (Fig. 7C).

## Discussion

Effective, safe, broad-spectrum antivirals are a necessity for treating infectious diseases. Here, we expand the broad-spectrum repertoire of an engineered banana lectin, H84T BanLec. It was unknown whether H84T BanLec would be effective against human herpesviruses, particularly VZV, HCMV, and HSV-1. In cell-based assays and adult human skin organ culture, H84T BanLec reduced viral spread, even when treatment was delayed. In the NuSkin humanized mouse model with adult skin xenografts, H84T BanLec prevented VZV spread when treatment was delivered subcutaneously before virus infection.

Understanding the mechanism of action is important for any antiviral compound or biologic. Here, we addressed the effectiveness of H84T BanLec against several human herpesviruses to prevent spread of VZV, HCMV, and HSV-1 in cultured cells and skin explants. Our working hypothesis is that H84T BanLec affects different stages of the virus life cycle for VZV and HSV-1 or HCMV. This is analogous to IAV, HIV and Ebola virus, where H84T BanLec primarily blocks different steps in the viral life cycle, including attachment, entry, fusion of the viral envelope in the endosome, and, specific to Ebola virus, steps downstream from entry but before budding^15,19,21,22^. Here, we showed that for VZV, H84T BanLec does not block attachment or entry. Instead, we found H84T BanLec interferes with post-entry steps in the viral replication process to prevent VZV spread. Remarkably, H84T BanLec caused an immediate decrease in VZV bioluminescence signal in infected skin one day after it was added up to 5 DPI. The replication time of VZV is approximately 24 h. These results support the cell-based studies, suggesting that H84T BanLec likely interacts with VZV-infected cells by inhibiting glycoprotein maturation, trafficking, virion assembly, or cell fusion. It will be important to address whether H84T BanLec interacts with VZV glycoproteins on the plasma membrane as they traffic from the trans-Golgi network to intracellular vesicles that are involved in envelopment^47^. For HCMV, H84T BanLec was most effective on extracellular virions. Time-of-addition assays showed that treating infected cells was no more effective than adding H84T BanLec to virions or cells during the infection process. It is plausible that H84T BanLec in the medium interacted with nascent virions as they were released after day 3. This is congruent with the results in skin organ culture. We also showed that infectious HCMV virions were made in the presence of H84T BanLec, but they did not appear to reach new cells to propagate the infection. Taken together, these results suggest that H84T BanLec does not interfere with HCMV virion synthesis, but that the mechanism of action is through prevention of virus attachment and entry. While the mechanism of action for HSV-1 was not tested in the current study, we found that H84T BanLec also reduced HSV-1 titers in skin. When treatment was initiated at the time of infection and when added 1 DPI, HSV-1 titers were significantly reduced, but the rapid replication time of HSV-1 meant that the compound lost effectiveness by 2 DPI. Thus, for HSV-1, we expect that H84T BanLec is also acting post-entry. More studies are needed to fully resolve this. Overall, it appears that H84T BanLec may have different mechanisms of action for alpha- and betaherpesviruses, consistent with our previous studies of other viruses^16,21,22^. It is important to note that the attachment, entry, and time of addition studies presented here were performed in HFFs. Others have shown that glycoprotein processing can vary dramatically between cell types (reviewed in^48^). This is well-established for HSV-1, where Pereira et al. showed that several HSV glycoproteins had strikingly different properties when the virus was grown in Hep-2 cells versus Vero cells^49^. Thus, it is possible that the viruses used in this study would have different glycosylation patterns if grown in different cells, potentially altering their ability to interact with H84T BanLec. To fully resolve the mechanism of action of H84T BanLec for each virus, it would be necessary to grow and evaluate the viruses with H84T BanLec in all relevant cell types.

The potent activity of H84T BanLec in skin, even when infection is established for several days, raises its potential for topical applications. H84T BanLec effectively blocked HIV infection with intravaginal application^15^, supporting use of H84T BanLec as a topical treatment to control or prevent virus infection and spread. We are exploring this idea for VZV and HSV-1 in the skin organ culture system. Preliminary studies are promising and showed that H84T BanLec formulated in cocoa butter or hydroxyethyl cellulose was highly effective at reducing VZV and HSV-1 spread (data not shown). Numerous potential applications for topical H84T BanLec exist: varicella and herpes zoster rashes, cold sores and genital blisters caused by HSV-1 and -2, or Kaposi sarcoma caused by the Kaposi sarcoma herpesvirus (KSHV). More studies are needed to develop H84T BanLec as a topical treatment, and the skin organ culture system will be useful.

An important consideration when using biologics like H84T BanLec is that exogenous proteins may be immunogenic. We have previously found that H84T BanLec elicited antibodies in mice when administered *ip*, yet this immune response did not diminish the effectiveness of subsequent treatments for IAV^21^. In the athymic nude mouse model used in this study, we did not assess immune responses to H84T BanLec since this mouse strain is immunodeficient and does not generate antibodies. The original BanLec molecule, which retains its mitogenic properties, altered cytokine levels and lymphocyte markers in mice^50^. We did not address these responses in our model because H84T BanLec is much less mitogenic compared to wild-type BanLec^15^ and, unlike the wild-type BanLec, does not lead to mitogenicity-induced ill effects in mice^21^. However, it may be possible in future studies to measure human cytokine responses in the SOC system. We have previously used the SOC system to analyze migrating immune cells, such as macrophages and dendritic cells, during HCMV infection^44^. In fact, we noticed that more cells appeared to migrate out of the skin pieces when H84T BanLec was added to the culture medium than in control cultures (data not shown). Thus, the SOC system could be used to address H84T BanLec effects on human immune cell responses and cytokine levels during an infection ex vivo. Further studies are necessary to fully elucidate the effects of H84T BanLec, including its immunomodulatory effects, on the host.

H84T BanLec was highly potent against VZV in cells and skin, even when added after infection, and so it was somewhat surprising that it did not prevent VZV spread in NuSkin mice when given either prophylactically or therapeutically by the *ip* route. In contrast, H84T BanLec was effective by the subcutaneous route, preventing VZV spread when given 6 h before and 4 and 8 DPI. It is not clear from this experiment if pretreatment or the sc route, or both, was the determining factor. Additional in vivo assays would be useful to determine the optimal route and timing of H84T BanLec delivery to prevent VZV spread. However, these assays are prohibitively costly to repeat as part of this project. It is likely that delivery by the *sc* route places H84T BanLec in proximity to the infected skin xenograft, which may be necessary for it to interact with the VZV-infected cells and free virions. Compounds delivered *sc* are absorbed and circulate through the bloodstream, which may also deliver H84T BanLec to the infected skin; in fact, H84T BanLec is moderately effective against IAV infection when given *sc* in the mouse^21^. On the other hand, compounds delivered *ip* and *iv* interact primarily with the liver and lungs, which may lower the amount that reaches the VZV-infected skin xenografts. Pharmacology studies in mice showed that H84T BanLec administered by *ip* injection distributed to the lung, liver, and spleen, and remained at high levels in the lung for longer than 72 h^21^. The large size of the H84T BanLec protein dimer, approximately 30 kDa, may also account for its retention in organs^19^. By comparison, cidofovir has a molecular weight of only 0.28 kDa. Thus, the large size of H84T BanLec and the route of administration may account for some of the differences between the skin organ culture and NuSkin mouse assays. However, additional pharmacokinetic studies and in vivo antiviral assays would be necessary to fully understand these discrepancies.

While biologics are becoming more mainstream treatments for cancer and infectious diseases, their route of administration is a major consideration. The size of biologics is another key factor in their development and clinical use [reviewed in^51^]. The large size necessitates intravenous delivery to reach their intended target. Efforts are underway to find alternative routes of delivery that are less invasive but still highly effective [reviewed in^52^]. H84T BanLec is effective when administered intranasally or subcutaneously in mice to treat IAV^15,21^, intraperitoneally to treat IAV and Ebola virus^21,22^, and intravaginally to prevent HIV transmission^15^. Alternatively, H84T BanLec could be administered orally, as have other plant lectins^14,53^. Griffithsin, a 13 kDa lectin, was effective against an HIV pseudovirus when given orally^54^, however virus neutralization was only monitored by rectal output. Finally, wild-type BanLec administered orally had no deleterious effects on mice^50^.

In this project, we expanded the spectrum of H84T BanLec to include the human herpesviruses VZV, HCMV, and HSV-1. We showed that H84T BanLec works differently to prevent VZV and HCMV spread, however more research is needed to fully resolve each mechanism of action. Additional research is also needed to optimize the routes of delivery, and timing of treatment in vivo. Despite this, our findings suggest that H84T BanLec is a promising antiviral and should be investigated further. Moreover, its proven effectiveness against viruses in different classes makes it likely that more viruses, or even abnormal cancer cells expressing surface N-linked glycans, will be targeted by H84T BanLec. It will be important to develop a variety of formulations for systemic and topical treatment to effectively treat this diversity of infections and diseases.

## Materials and methods

### Cells and viruses

Human foreskin fibroblasts (HFFs; CCD-1137Sk; American Type Culture Collection [ATCC], Manassas, Virginia), adherent retinal pigmented epithelial cells (ARPE-19; CRL-2302; ATCC), human embryonic lung fibroblasts (HEL-299 s; CCL-137; ATCC), and adult African green monkey kidney epithelial cells (Vero cells; ATCC) were used. Vero cells were kindly provided by Dr. Eain Murphy (SUNY Upstate Medical University, Syracuse, NY). Cells were grown in Dulbecco’s Modified Eagle Medium with 4.5 g/L-glucose, L-glutamine, and sodium pyruvate (DMEM, 1X, Corning, Manassas, VA). DMEM was supplemented with up to 10% heat-inactivated fetal bovine serum (Benchmark FBS; Gemini Bio Products, West Sacramento, CA), penicillin–streptomycin (5,000 IU/ml), and amphotericin B (250 μg/ml). HFFs and HELs were used up to passage 18, while ARPE-19 and Vero cells can be used for 30 + passages.

VZV-ORF57-Luc, kindly provided by Dr. Paul Kinchington (University of Pittsburgh, Pittsburgh, PA), expresses firefly luciferase inserted in frame after the ORF57 coding sequence and separated by a T2A ribosomal skip sequence^44^. VZV-ORF57-Luc was derived from the parental Oka strain^55^ and several mutations in the genome sequence were corrected. VZV-ORF57-Luc was passaged up to 10 times in HFFs or ARPE-19 cells. HCMV-fLuc-eGFP, kindly provided by Dr. Eain Murphy (SUNY Upstate Medical University, Syracuse, NY), was derived from the TB40/E variant of the TB40 clinical isolate^56,57^. HCMV-fLuc-eGFP expresses firefly luciferase (fLuc) and enhanced green fluorescent protein (eGFP)^44^. Firefly luciferase is regulated by the true late UL18 promoter and replaces UL18, a nonessential glycoprotein. Enhanced GFP is regulated by the constitutively active SV40 promoter and is inserted between US34 and TRS1. HCMV-fLuc-eGFP is derived from a BAC and has been passaged up to 5 times in HELs. HSV-1 R8411 is derived from F strain and expresses firefly luciferase regulated by the ICP27 promoter (kindly provided by Dr. Bernard Roizman, University of Chicago, Chicago, IL). HSV-1 R8411 was propagated in Vero cells. All viruses were stored at -80 °C. Virus inoculum varies and was selected based on location of infection and density of infectable cell types.

### Drug and compound formulation

H84T BanLec was prepared from a lyophilized powder and reconstituted in sterile water at 20 mg/mL and stored at − 80 °C. Cidofovir, acyclovir, and ganciclovir were prepared from powder in dimethyl sulfoxide (DMSO) or sterile water and stored at − 20 °C. Stock compounds were diluted in complete tissue culture medium for cell-based and SOC assays. For mouse studies, H84T BanLec was diluted in saline to achieve a concentration of 50 mg/kg/day in 0.1 mL and stored at − 80 °C. For mouse studies, cidofovir was also prepared in saline to achieve a concentration of 20 mg/kg/day in 0.1 mL and stored at 4 °C.

### Preparation of adult human skin

Skin was obtained from reduction mammoplasty and complete mastectomy surgeries in accordance with approved Institutional Review Board procedures and protocols at SUNY Upstate Medical University (IRB #1140572, SUNY Upstate Institutional Review Board, Syracuse, NY). All tissue was ethically obtained in accordance with institutional, local, state and national guidelines. The entire experimental procedure has been reviewed and approved by the Institutional Review Board at SUNY Upstate Medical University (Syracuse, NY). Written informed consent to obtain excess skin tissue was given by healthy adults over 18 years old. Skin was collected in saline within 2 h of surgery and processed for use per established protocol^44,58^. Briefly, whole thickness skin was cleaned with povidone-iodine and 70% EtOH, rinsed with tissue culture medium, stretched across a Teflon-covered foam cylinder, and secured with pins. Skin was thinned with a Weck (skin grafting) knife fitted with a 0.028″ Goulian guard, yielding thinned skin approximately 700 microns thick. Thinned skin was cut into approximately 1-cm^2^ pieces for skin organ culture studies or implantation in mice.

### Efficacy and cytotoxicity analysis

Compounds were evaluated for antiviral activity against VZV, HCMV, and HSV-1 using previously established dose response assays^59^. Briefly, cells were seeded in tissue culture treated 24-well plates 1–3 d prior to infection. Cells were infected with cell-free virus and H84T BanLec was added at varying concentrations at the time of infection (0 HPI) or after virus attachment and entry (2 HPI) at 37 °C. Control compounds (cidofovir, ganciclovir, and acyclovir) were added at 2 HPI. For VZV assays, confluent ARPE-19 cells or HFFs were infected at MOI 0.01 for 3 d. For HCMV assays, confluent HFFs were infected at MOI 0.05 for 7 d. For HSV-1 assays, sub-confluent Vero cells were infected at MOI 0.01 for 48 h. After the infection period, the medium was replaced by D-luciferin (300 µg/mL in PBS) for 40 min at 37 °C prior to scanning. Virus spread was measured by bioluminescence (total flux; photons/s/cm^2^/steradian) using the IVIS® 50 instrument (Caliper Life Sciences/Xenogen, Hopkinton, MA). Virus yield was calculated as the total flux at each concentration divided by the average total flux for untreated wells. The 50% effective concentration (EC_50_) for each compound and condition was calculated using Graphpad (GraphPad Software, San Diego, California, www.graphpad.com).

Cytotoxicity was measured using a neutral red assay (NR) as previously described^43,59^. Cells were added to 96-well plates at the same densities and timing used to determine the EC_50_. Cells were treated with compounds for either 2 d (Vero) or 3 d (HFFs and ARPE-19 cells). Staurosporine was used as a positive control for cell death. Results from the neutral red assay were used to calculate the 50% reduction in cell viability (CC_50_). The EC_50_ and CC_50_ were used to calculate the selectivity index (SI) for each compound against each virus.

### H84T BanLec virus life cycle assays

Cell-free virus and confluent HFFs were used for all experiments. H84T BanLec was used at concentrations near the EC_90_: 100 nM for VZV and 2 µM for HCMV. To treat virions, VZV or HCMV and H84T BanLec were incubated together on ice for 1 h, diluted in tissue culture media (1:50 for VZV, 1:1000 for HCMV) and added to HFFs. To study attachment, cells were treated with H84T BanLec for 1 h on ice, then treatment was removed and replaced with the virus inoculum for 1 h on ice. Virus inoculum was removed and replaced with fresh media. To study attachment and entry, H84T BanLec and virus were combined and added to HFFs at 37 °C for 1 h, then removed. To study entry, HFFs were infected with virus on ice for 1 h, the virus was removed and H84T BanLec was added at 37 °C for 30 min. Excess virions and H84T BanLec were removed with a citrate buffer wash (pH 6.0; Millipore, Billerica, MA), and media was replaced. VZV-infected cells were scanned by IVIS at 24 h post-infection and HCMV-infected cells were scanned at 7 d post-infection. For time-of-addition studies, HFFs were infected with VZV or HCMV for 1 h at 37 °C, then excess virions were removed, and media was replaced. For VZV cultures, H84T BanLec was added to infected cells at 1, 2, 4, 8, or 24 h post-infection, and cells were scanned by IVIS at 48 h post-infection. For HCMV, H84T BanLec was added to infected cells 1, 24, 48, 72, or 96 h post-infection, and cells were scanned at 7 d post-infection. Unless otherwise indicated, plates were incubated at 37 °C with 5% CO_2_.

### Skin organ culture

Adult human skin was cultured on NetWells, which are designed to hold tissue at the air–liquid interface, exposing the dermis to tissue culture media while the epidermis is exposed to air. Skin was cultured on NetWells with DMEM supplemented with 4% heat-inactivated fetal bovine serum, penicillin–streptomycin, and amphotericin B. Uninfected skin was incubated at 35 °C in a humidified incubator with 5% CO_2_. For VZV skin organ culture, VZV-ORF57-Luc was grown in ARPE-19 cells to > 80% CPE (cytopathic effect), and infected cells were harvested with trypsin/EDTA and sonicated to create a cell-free virus inoculum. Skin was inoculated with VZV (1 × 10^4^–10^5^ pfu/mL) by scarification using a 27-gauge needle^43,44,60^. Skin was incubated at 35 °C for 2 h to absorb the virus inoculum, then returned to NetWells. If applicable, treatment was added to the medium at this time. For HSV-1 skin organ culture, thinned skin was treated with a derma roller (0.5 mm tines) prior to cutting it into 1-cm^2^ pieces. A cell-free inoculum of HSV-1 R8411 prepared in Vero cells was added to each skin piece (1 × 10^5^ pfu/piece). As with VZV, skin was placed at 35 °C for the inoculum to absorb prior to transferring to NetWells and starting treatment. For HCMV skin organ culture, cell-free HCMV-fLuc-eGFP grown in HEL cells (5 × 10^6^ pfu/piece) was inoculated by intradermal injection using a 27-gauge needle^44^. Skin was placed at 37 °C for 1–2 h to allow for virus to absorb, and then transferred to NetWells where treatment was started, if applicable. Skin was imaged using the IVIS® 50 instrument to measure bioluminescent signal. For imaging, skin was transferred to 24-well black plates with black bottoms and submerged in D-luciferin (300 µg/mL in PBS) for 40 min prior to scanning. After scanning, skin was returned to NetWells. Culture medium and compounds were refreshed daily (HSV-1) or every other day (VZV and HCMV). After the final day of imaging, infected skin was divided: one half was fixed in 4% paraformaldehyde and stored at 4 °C for future histopathological analysis, and the other half was processed for virus extraction and plaque assay.

### Virus extraction and quantification

To extract HCMV from skin, tissue was minced, placed in complete tissue culture medium in Eppendorf tubes with glass beads, and put through three rapid-freeze thaw cycles with a 30 s vortex between cycles and a 2 min vortex after the final cycle^44^. The mixture was centrifuged at low speeds to remove tissue debris, and the supernatant was aliquoted and stored at − 80 °C. To quantify HCMV, supernatant was applied to HFFs in 96-well plates to perform a TCID_50_ per standard protocol. The presence of infectious virus was determined based on GFP signal 7–10 days post-inoculation. To extract HSV-1 from skin, tissue was cut in half, placed in complete tissue culture medium and subjected to three rapid freeze–thaw cycles followed by a quick vortex. Supernatant was aliquoted and stored at − 80 °C. To quantify HSV-1, supernatant was applied to Vero cells in 6-well plates for 1 h to allow for attachment and entry. Then, an overlay of 1.25% methylcellulose in complete tissue culture medium (10% FBS) was applied to all monolayers. Plates were incubated until well-defined plaques were formed, approximately 3–5 days post-inoculation, the methylcellulose overlay was removed, cell monolayers were stained with crystal violet, and plaques in each well were counted.

### Histopathology

Skin was fixed in 4% paraformaldehyde and sent to a commercial lab for processing by standard operating procedure with a fully automated workflow (HistoWiz Inc., histowiz.com, New York, NY). Samples were embedded in paraffin and sectioned at 5 μm. Hematoxylin and eosin (H&E) staining was performed by standard operating procedure, and sections were dehydrated and film coverslipped using a TissueTek-Prisma and Coverslipper (Sakura Finetek USA, Inc., Torrance, CA). Whole slide scanning (40x) was performed on an Aperio AT2 (Leica Biosystems, Buffalo Grove, IL).

### Animal procedures

All animal procedures were performed with approved protocols under the guidance of the Institutional Animal Care and Use Committee (IACUC) at SUNY Upstate Medical University and in accordance with all state and federal laws and regulations. Furthermore, the entire experimental procedure has been reviewed and approved by the IACUC at SUNY Upstate Medical University. Additionally, all animal studies were performed and reported in accordance with ARRIVE guidelines (https://arriveguidelines.org/). Adult human skin was placed subcutaneously above the left flank in athymic nude mice (male, age 5–6 weeks, CR ATH Ho; Crl:NU(NCr)-*Foxn1*^*nu*^, Charles River, Wilmington, MA). Single, full thickness xenografts were placed in each mouse per established protocol^44^. Three to four weeks post-implantation, fully vascularized xenografts were inoculated with VZV-ORF57-Luc (cell-associated in HFFs, 1 × 10^5^–10^6^ pfu/mL, 60 µL) by intradermal injection and scarification^44^. Virus growth was monitored with daily bioluminescence imaging (IVIS® 200, Caliper Life Sciences/Xenogen, Hopkinton, MA). Test compounds or cidofovir in saline were administered by subcutaneous (*sc*) or intraperitoneal (*ip*) injection. H84T BanLec was administered to one group 6 h pre-inoculation (HPI), 4 days post-inoculation (DPI), and 8 DPI, and to another group on 3 and 7 DPI. Cidofovir was administered daily 3–9 DPI. Mice were weighed and observed daily for signs of distress.

### Bioluminescence imaging

Bioluminescence imaging was performed per established protocol^43^. Cell cultures and skin organ cultures were scanned with the IVIS® 50 and images acquired for 30 s – 5 min based on pixel saturation and signal intensity. Mice infected with VZV were scanned with the IVIS® 200 and images acquired for 1–5 min depending on pixel saturation. The extent of virus spread was measured as Total Flux (photons/sec/cm^2^/steradian) in a region-of-interest (ROI) encompassing each skin piece. Total flux was used to determine fold change, which was calculated as the daily total flux divided by the lowest total flux value (DPI 1 for SOC; DPI 2 or 3 for mice).

### Statistics

All calculations and graphs were made using GraphPad Prism (Graph-Pad Software, San Diego, CA, www.graphpad.com). The 50% effective concentration (EC_50_) and 50% cytotoxic concentration (CC_50_) values were calculated using nonlinear regression analysis to fit a dose vs. response curve (F constrained to 50). Data from skin organ culture and mouse experiments were analyzed using one-way ANOVA with Dunnett’s or Dunn’s post hoc test. Data from virus quantification were analyzed using either a one-way ANOVA with Dunnett’s post hoc test or a Student’s t-test between vehicle and treatment groups. *p* ≤ 0.05 was considered statistically significant.
